# “Unsteady Gait”: An Uncommon Presentation and Course of Malignant Melanoma in Terminal Ileum—A Case Report and Review of Literature

**DOI:** 10.1155/2013/958041

**Published:** 2013-11-28

**Authors:** Satya Allaparthi, Khalid A. Alkimawi

**Affiliations:** ^1^Department of Medicine, Saint Vincent Hospital, 123 Summer Street, Worcester, MA 01608, USA; ^2^Department of Gastroenterology, St Elizabeth's Medical Center and Tufts Medical Center, Boston, MA 02111, USA

## Abstract

Malignant melanoma within the gastrointestinal tract is an uncommon neoplasm that is usually metastatic in origin, with primary melanomas being relatively uncommon. Embryologically melanocytes normally exist in the esophagus, stomach, small bowel, and anorectum and this theory supports the primary melanoma of the gastrointestinal tract that has been confirmed for lesions occurring through several published reports. However, most patients with brain metastases from malignant melanoma are diagnosed after treatment for known extracranial metastases and have poor outcomes. Our case is unique in that we discuss an unusual case of 69-year-old female patient presented with unsteady gait as the first symptom of disease and where the presumed primary lesion later was found in the terminal ileum on colonoscopy. Treatment consisted of surgical removal of the terminal ileal lesion with chemotherapy, whole-brain radiotherapy, and cyberknife radiosurgical procedure. Patient was in remission for more than 14 months and later succumbed to disease. Despite the advances in therapeutic options, prognosis for patients with melanoma brain metastases remains poor with a median survival time of six months after diagnosis.

## 1. Introduction

Next to lung cancer, malignant melanoma is the most frequent cause of brain metastasis. In a large series from the Metropolitan Detroit Cancer, the cumulative incidence of melanoma brain metastasis is <10% and usually develop late in the course of the disease [1, 2]. Metastatic spread of tumor cells detached from melanoma into the central nervous system (CNS) occurs haematogenically since lymphatic drainage is absent in the brain. The blood-brain barrier is usually intact in metastases that are smaller than 0.25 mm in diameter because melanoma micrometastases are common in the brain and patients can harbor numerous metastases in the brain without any neurological deficits [3, 4]. Furthermore, while melanoma can present in the brain as the first site of metastasis, it is more common for brain metastasis to present later in the course of disease, most often acting as a harbinger of terminal disease. The course of disease is typically characterized by rapid extra cranial progression and short overall survival time despite various local and systemic treatment approaches. While surgery and radiotherapy interventions can prolong the disease-free interval when solitary, large metastases in the brain are found early in the course of melanoma metastasis; these treatments provide only short-term, but nevertheless important, palliation in patients with multiple brain lesions. We report an unusual case where in our index patient presented with unsteady gait and blurry vision as the first symptom and on further workup was found to have a presumed primary malignant melanoma in the terminal ileum.

## 2. Case Report

**Figure 1:**
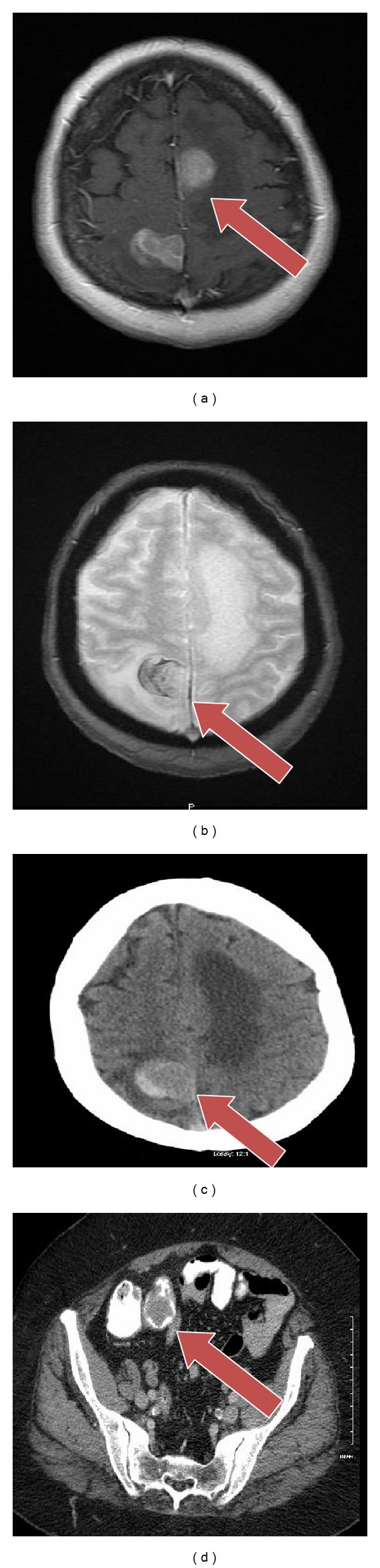
(a), (b), (c) Arrows showing MRI of brain with and without gadolinium with metastatic lesions in parietal and occipital region. (d) Showing CT scan of abdomen and pelvis with filling defect in terminal ileum.

**Figure 2:**
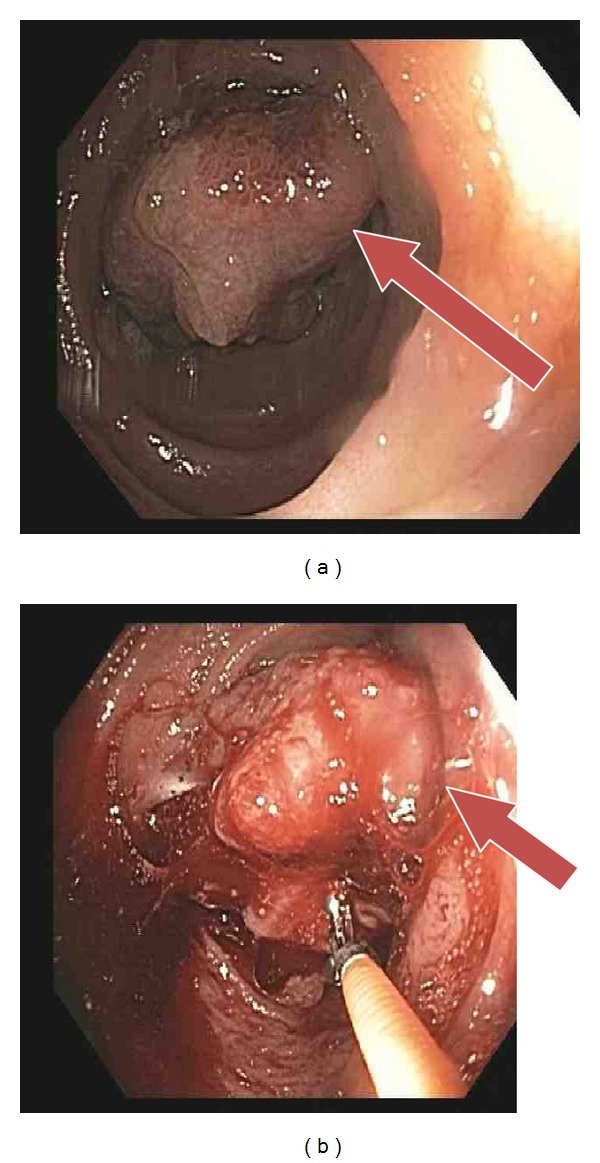
(a) Showing colonoscopy view of mass in the terminal ileum. (b) Showing biopsy of the lesion.

**Figure 3:**
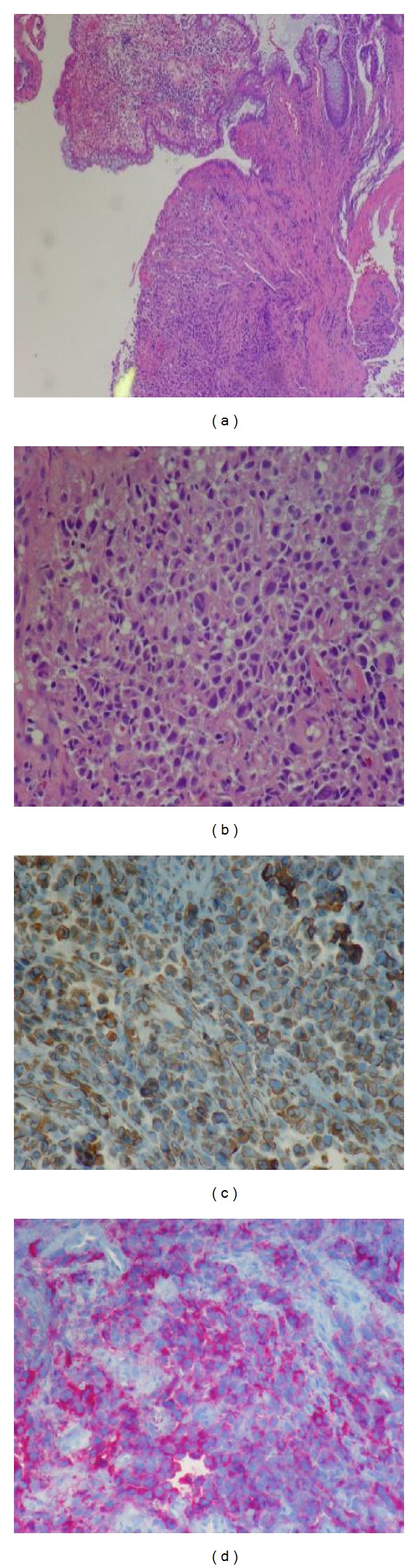
(a) Hematoxylin-eosin stain of melanoma in terminal ileum. (b) Hematoxylin-eosin stain of melanoma in terminal ileum, original magnification ×400. (c) Specimen stained with c-kit immunohistochemistry, original magnification ×400. (d) Specimen stained with HMB 45+ antibody original magnification ×400.

A 65-year-old Caucasian female with prior history of hypertension and hyperlipidemia presented with unsteady gait, weakness in her left leg, and blurry vision. Patient had intermittent symptoms for last few weeks; however, she did not seek any medical attention. In view of her persistent symptoms, she was evaluated by medical team for a possible neurological cause. Review of systems was otherwise unremarkable. She takes simvastatin and hydrochlorothiazide for dyslipidemia and hypertension, respectively. There were no other significant past medical or surgical history to contribute for the presenting complaints. Clinical examination revealed decrease in power in left lower extremity with preserved reflexes. All routine blood tests were within normal limits. A magnetic resonance imaging (MRI) (Figures 1(a), 1(b), and 1(c)) scan of the brain revealed four enhancing brain masses largest in the right parietal lobe and others in right frontal and temporal lobe and left parietal lobe, respectively. Further staging workup included examinations of the eyes, head, and neck mucosa, total skin, gynecological evaluation, bone scintigraphy, and computed tomography (CT) scans of abdomen and pelvis (Figure 1(d)) that showed a filling defect in terminal ileum as the only pathological finding. Colonoscopy revealed a partially pigmented polypoid lesion in the terminal ileum and a biopsy done was suggestive of malignant melanoma (Figure 2). Further pathological evaluation revealed it as epithelioid variant with ulceration and negative for BRAF oncogene (Figure 3). Patient underwent resection of terminal ileum with end-to-end anastomosis. After resection she underwent chemotherapy with ipilimumab and dacarbazine every three weeks for 4 cycles. After chemotherapy she received 30 Gys of whole-brain radiotherapy (WBRT) for ten sessions. Followed by this she had a Cyberknife, (Accuray Inc., Sunnyvale, CA) an image-guided robotic radiosurgery. The treatment procedure included CT image acquisition based on skull-bone landmarks, planning, and radiation dose delivered at 18–20 Gys based on size of lesion for three treatments. Despite aggressive treatment patient succumbed to the disease in 14 months. Our index case accounts for the less than 2% of cases reported in literature as a rare presentation of primary malignant melanoma in the gastrointestinal tract (GI) without evidence of any primary skin lesion or any other sites with multiple brain metastases.

## 3. Discussion

Malignant melanoma of the skin or epithelia is known to metastasize to the intestines. Primary melanomas of the GI tract, however, are a very rare entity. They are rarely diagnosed at an early stage, tend to be more aggressive, and are associated with a poor prognosis. Neurological symptoms as the first sign of malignant melanoma are relatively uncommon, as is the inability to identify the primary tumor in patients with brain metastases from this disease [5].

The common feature of all melanomas is the cell of origin, the melanocytes. Melanocytes are usually absent in the small bowel and colon. However, various authors postulated different theories that included origin from schwannian neuroblast cells associated with the autonomic innervations of the gut as by Mishima [6]. Amar et al. reported origin of melanoma in melanoblastic cells of the neural crest which migrate to the distal ileum through omphalomesenteric canal or in APUD cells [7] which can undergo neoplastic transformation and produce tumors such as carcinoids or gastrinomas. According to the APUD theory, the ileum, which represents the distal end of the umbilical mesenteric canal, should be the most common site of primary malignant melanoma within the small intestine [8]. However, some researchers suggest that primary melanoma of the small bowel does not exist as a separate clinical entity and that all small bowel melanomas are metastatic lesions from unknown or regressed primary cutaneous melanoma [9]. When considering histo-pathological features alone, a clear distinction between primary intestinal melanoma and intestinal metastatic deposits is complex. Metastatic melanomas of the small bowel were classified into cavitary, infiltrating, exoenteric, and polypoid based on radiological examinations and are always not distinct [10]. The polypoid pattern, equally distributed between the jejunum and ileum, is the most common manifestation of metastatic melanoma to the small bowel. Histological features of metastasized intestinal melanoma that develop after spontaneous regression of primary cutaneous melanoma include lymphocytic infiltration of the dermis with melanophages, vascular proliferation, and reparative fibrosis [11]. Amersi et al. [12] in their study showed that functionally active CCR9 on melanoma cells facilitates metastasis to the small intestine. The CCR9-CCL25 axis may explain the high incidence of melanoma metastasis to this specific location like a “homing receptor” for melanoma of the small bowel. The time frame period between diagnosis of primary malignant melanoma and the identification of metastases at a gastrointestinal level varies between 2 and 180 months and most of them is detected only during autopsy [13].

Melanoma of the small bowel has very vague clinical presentation varying from completely asymptomatic presentation to myriad of symptomatic presentations that include chronic abdominal pain 17–64%, occult or gross bleeding 26–84%, and weight loss 10–47% [13]. Rarely it can present as acute surgical emergency due to intestinal obstruction or intestinal intussusceptions and, rarely, to bowel perforation. A handful cases of bowel perforation and intussusceptions were reported in medical literature secondary to metastatic melanoma [14–18]. Rarity of occurrence combined with vague clinical presentations poses a clinical dilemma in evaluating patients with small bowel melanoma. Different imaging techniques like CT scan and capsule endoscopy may give a suspicion of intestinal neoplasm; however, the final diagnosis can be obtained only after endoscopic or colonoscopic guided biopsy. The sporadic nature and the small numbers of patients reported in the literature with a primary small bowel melanoma pose an endoscopic and surgical dilemma for the gastroenterologists and surgeons, respectively.

A wide intestinal resection along with the resection of the mesentery with lymph nodes remains the treatment of choice as intervention improved survival significantly, especially when resection was complete on microscopic examination as reported by Ollila et al. [19]. In their study, the median survival period after complete surgical resection of GI metastases was 48.9 months versus 5.4 months after incomplete resection and the 5 years survival rate was 41% after complete resection.

Our index case had a polypoidal mass in terminal ileum that was detected shortly after diagnosis of the brain lesions that was confirmed on colonoscopy and biopsy. As reported in the literature, CNS metastases occur in 10 to 40% of melanoma patients in clinical studies and up to 90% in autopsy studies [20]. At five years, the cumulative risk for patients with melanoma to develop CNS metastases corresponds to about approximately 7% [21]. Seventy-one percent of the primary lesions are invasive lesions with mean greater than thickness of 3.5 mm. Saha et al. in their studies showed a prevalence of about 5% in patients with multiple melanoma metastases that have more than five intracerebral metastatic lesions [22].

Galicich [23] found an association between the size of the cerebral metastatic lesion from malignant melanoma and clinical parameters characteristic of tumor behavior. The clinical course of disease and their response to treatment can be predicted based on the size of the lesion. Lesions are classified as smaller than 1 cm (group A), between 1–4 cm (group B), and bigger than 4 cm (group C), respectively. Group B lesions are the most common, independent of the site of the primary tumor, except for patients with rectal melanoma. Group C metastases are the least common and are usually solitary. Asymptomatic patients usually have group A metastases, whereas those with nonspecific complaints or behavioural changes usually have group B metastases. Solitary lesions usually belong to group B or C, whereas multiple lesions belong mainly to group A or B. Based on this classification, our index patient fits into the category of group B metastatic lesions.

In patients with metastatic brain lesions, radiotherapy plays an important role in palliative treatment. Fife et al. [5] in their study of 1137 patients reviewed the outcomes of patients who underwent surgery, whole brain radiotherapy (WBRT), and combined modalities. Patients with a single brain metastasis managed with surgical resection plus WBRT have a 2-year survival rate of 20–25%. Other prognostic factors included younger age, long disease-free interval, and no concurrent extracranial metastases. In our case, patient underwent intestinal resection with limited lymph node dissection followed by chemotherapy, WBRT, and gamma knife radio surgery (GKRS). GKRS for melanoma brain metastases was reported to result in 1-year local control in 49% and overall survival in 25% of the patients, with survival being dependent on the score index for radio surgery (SIR) [24]. Wong and Coit [25] in their study reported that in patients with metastatic melanoma a procedure performed with palliative intent in appropriately selected patients usually experience reliable relief of symptoms and improved quality of life. Improved survival after a complete resection with curative intent is often predicted by good performance status, longer disease-free interval, limited extent of metastatic disease, and less aggressive tumor biology. Our index patient had multiple brain metastases who underwent multiple radio surgical procedures as a palliative option for symptomatic relief and survived for 14 months.

## 4. Conclusion

In conclusion, our case highlights a unique presentation and course of metastatic melanoma and illustrates that patients with multiorgan melanoma manifestations may benefit from the repeated use of effective local therapeutic approaches for palliative and in a short term. Further studies would be needed to evaluate long-term outcomes.
